# Hypocalcemia is associated with adverse outcomes in patients hospitalized with COVID-19

**DOI:** 10.1007/s12020-022-03239-w

**Published:** 2022-11-09

**Authors:** Alessandro Minasi, Aikaterini Andreadi, Alessio Maiorino, Luca Giudice, Sofia De Taddeo, Ilenia D’Ippolito, Ilaria de Guido, Rossella Laitano, Maria Romano, Valeria Ruotolo, Andrea Magrini, Nicola Di Daniele, Paola Rogliani, Alfonso Bellia, Davide Lauro

**Affiliations:** 1 Department of Systems Medicine, University of Rome “Tor Vergata”, Rome, Italy; 2 Division of Endocrinology and Diabetes, University Hospital Fondazione Policlinico Tor Vergata, Rome, Italy; 3 Department of Experimental Medicine, University of Rome “Tor Vergata”, Rome, Italy; 4 Division of Respiratory Medicine, University Hospital Fondazione Policlinico Tor Vergata, Rome, Italy; 5 Department of Biomedicine and Prevention, University of Rome “Tor Vergata”, Rome, Italy

**Keywords:** COVID-19, Hypocalcemia, Total serum calcium, Vitamin D

## Abstract

**Purpose:**

Calcium ions are involved in the regulation of several cellular processes and may also influence viral replication. Hypocalcemia has been frequently reported during infectious diseases and in critically ill patients, including also COVID-19 patients, significantly related with the pro-inflammatory state and mortality. The aim of this study is to investigate the prevalence of hypocalcemia at admission in patients hospitalized for COVID-19 (*Coronavirus disease* 2019) and to evaluate association of hypocalcemia with in-hospital COVID-19 outcomes.

**Methods:**

Retrospective analysis on 118 consecutive patients, hospitalized for COVID-19 between March and May 2020. Clinical characteristics, inflammation markers, biochemical routine and mineral metabolism parameters at admission were collected. Hypocalcemia was defined as total serum calcium <2.2 mmol/L. Population was stratified by tertiles of total serum calcium. Primary outcome was the composite of in-hospital death or admission to intensive care unit (ICU). Secondary outcomes included in-hospital death, admission to ICU and need for non-invasive ventilation as separate events. Associations were tested by logistic regression and Cox-regression analysis with survival curves.

**Results:**

Overall prevalence of hypocalcemia was 76.6%, with just 6.7% of patients reporting levels of 25-(OH)-vitamin D > 30 ng/ml. Total serum calcium was inversely related with selected inflammatory biomarkers (*p* < 0.05) and poorer outcome of COVID-19 during hospitalization. Lower tertile of total calcium (≤2.02 mmol/L) had increased risk of in-hospital mortality (HR 2.77; 1.28–6.03, *p* = 0.01) compared with other groups.

**Conclusion:**

Total serum calcium detected on admission is inversely related with proinflammatory biomarkers of severe COVID-19 and is useful to better define risk stratification for adverse in-hospital outcome.

## Introduction

In December 2019, in China, SARS-CoV-2 (*Severe Acute Respiratory Syndrome Coronavirus* 2) was identified as the causative agent of a cluster of pneumonia of unknown origin. The infection, due to its rapid global spread, in March 2020, was declared by the WHO (World Health Organization) an international public health emergency, results in COVID-19 (*Coronavirus disease* 2019) characterized by extensive involvement of the respiratory system, from mild and self-limiting forms up to severe viral pneumonia with respiratory failure, multi-organ dysfunction and death. Although the majority of affected patients shows only moderate symptoms, especially fever and dry cough, the disease has a non-negligible lethality with a substantial proportion of subjects requiring hospitalization and ventilatory assistance [1, 2]. Several authors reported a high prevalence of hypocalcemia among COVID-19 patients at the time of the first in-hospital evaluation and this disorder seems to be one of the biochemical distinctive features of the disease [3–6]. Moreover, a typical osteo-metabolic phenotype, characterized by a high rate of hypocalcemia, hypovitaminosis D and high prevalence of vertebral fractures, has been observed in these patients and appears to be clinically relevant for its negative effects on disease course [7]. Calcium ions (Ca^2+^) act as second messengers within mammalian cells involved in the regulation of almost all cellular processes and also play a fundamental role in viral replication mechanisms, as specifically described for SARS-CoV (*Severe Acute Respiratory Syndrome Coronavirus*), MERS-CoV (*Middle Est Respiratory Syndrome Coronavirus*) and Ebola viruses [8–11]. Moreover, some studies reported low levels of serum calcium in hospitalized patients during SARS, Dengue and Ebola infections [12–14]. Although there is a complex regulatory system deputy to maintain the extracellular concentrations of ionized calcium within a narrow physiological range, hypocalcemia is a common phenomenon in critically ill patients [15]. A prominent role within this system is played by the calcium-sensing receptor (CaSR), a G protein-coupled receptor expressed in all key tissues involved in the regulation of calcium metabolism, including parathyroid cells, C parafollicular thyroid cells, kidneys, intestine and bone that perceives even minimal changes in blood levels of Ca^2+^ in order to restore the values in the physiological range [16]. Several studies investigated the predictive estimate of low serum calcium concentrations in critically ill patients and most of them found that hypocalcemia was associated with increased mortality [17–20], with proinflammatory cytokines playing a central role in calcium homeostasis alterations [21], and these findings were confirmed also in COVID-19 patients [22–24]. Aim of our study was to investigate the prevalence of hypocalcemia at the time of the first in-hospital evaluation among patients admitted for symptomatic and confirmed SARS-CoV-2 infection. Second, we evaluated the association of low serum calcium levels with inflammatory biochemical markers and clinical outcomes (in-hospital death, need for non-invasive mechanical ventilation and intensive care unit admission), in order to confirm its usefulness as marker of severe COVID-19 course, as reported in previous studies.

## Methods

We conducted a retrospective analysis on 118 consecutive adult patients, who had presented to the Emergency Room (ER) of University Hospital ‘Fondazione Policlinico Tor Vergata’ (Rome), between March and May 2020, during the first COVID-19 emergency in Italy and subsequently referred to the Pulmonary and Endocrinology Unit after the confirmation of SARS-CoV-2 infection. Eligibility criteria for being included in the present analysis were as follows: (i) laboratory-confirmed diagnosis of SARS-CoV-2 infection by real-time PCR test in respiratory specimens; (ii) clinical/radiological manifestations of disease on chest computed tomography (namely ground-glass opacity and/or crazy paving); (iii) estimated glomerular filtration rate (eGFR) > 30 ml/min/1.73 m^2^ according to CKD-EPI formula; (iv) no history of diseases or drugs known to affect mineral metabolism, as calcium or vitamin D supplements, systemic glucocorticoids, estrogens or anti-convulsant medications. After exclusion of seven patients because of impaired renal function, the remaining study population was divided into tertile categories according to serum calcium values at admission (≤2.02 mmol/L, 2.02–2.15 mmol/L and ≥2.15 mmol/L) for the comparison of the main clinical characteristics among the groups. All hospitalized patients received standard treatment according to local practice at the time of data collection, including mild to invasive respiratory support, nutritional, antimicrobial and other supportive therapies as needed. Specific therapies for SARS-CoV-2 included empirical off-label administration of hydroxychloroquine, low-molecular weight heparin, antivirals (lopinavir/ritonavir or darunavir/ritonavir) and tocilizumab.

Hospital clinical records were used as data source for extraction and data analysis. The following clinical data were analyzed: age, gender, ethnicity, weight, height, BMI, time (days) between onset of symptoms and presentation to the Emergency Room (ER), length of hospital stay, ongoing medications prior to admission, presence of diabetes mellitus, hypertension, cardiovascular diseases, chronic obstructive pulmonary disease (COPD), off-label administration of hydroxychloroquine, antivirals (lopinavir/ritonavir or darunavir/ritonavir) and tocilizumab during hospital stay. Biochemical data at the time of admission included C-reactive protein (CRP), procalcitonin, interleukin-6 (IL-6), D-dimer, fibrinogen, creatinine, eGFR, 25-OH-vitamin D (25-OH-D), parathyroid hormone (PTH), serum calcium, albumin-adjusted calcium according to the formula “*albumin-adjusted calcium (mmol/L)* = *total calcium (mmol/L)* + *0.02 [40 – albumin (g/L)]*”, serum phosphorus and magnesium. Hypocalcemia was defined as total serum calcium <2.2 mmol/L (8.8 mg/dL). Patients were classified as having cardiovascular diseases if coronary heart disease or arrhythmias or heart failure were present, according to reported medical history or ongoing medications. Presence of diabetes mellitus was assessed if patients were taking glucose-lowering medications or had HbA_1c_ levels ≥48 mmol/mol. As well, presence of hypertension and COPD were defined if patients were taking anti-hypertensive or bronchodilators, respectively. The composite of in-hospital death or admission to the intensive care unit (ICU) with need for endotracheal intubation was considered as the primary outcome measure to define severe course of COVID-19. Secondary outcomes included isolated in-hospital death, admission to ICU and need for non-invasive ventilation (NIV).

The present study has a retrospective design and therefore did not interfere with the course of medical management. The protocol was designed in accordance with the principles of Declaration of Helsinki for studies in humans and approved by local Ethics Authorities. All patients gave their informed consent to participate.

### Statistical analysis

Statistical analysis was performed with the SPSS Statistics 26 software. Means ± SD or median (interquartile range) were used as descriptive statistics for normally distributed or skewed continuous variables, respectively. Categorical variables were expressed as absolute and percent values. All quantitative variables were tested for normality distribution using the Kolmogorov–Smirnov test. Differences in normally distributed continuous variables among tertile categories of serum calcium were assessed using the ANOVA test, whereas the Kruskal–Wallis test was used in case of skewed variables. Post-hoc multiple comparisons were assessed by applying Bonferroni correction for ANOVA or Kruskal–Wallis test as needed. Differences in proportions of discrete traits were assessed using *χ*² test and Fisher’s exact test. Associations of variables with the selected outcomes were first analyzed using univariate regression analysis, and odds ratio (OR) with 95%CIs were given to measure strength of the relationship. The following parameters were initially tested: age, gender, days to ED admission, length of hospital stay, BMI, serum calcium, phosphorus, magnesium, 25-OH-D, PTH, CRP, procalcitonin, IL-6, D-dimer, fibrinogen, creatinine, categories of serum calcium, presence of diabetes mellitus, hypertension, cardiovascular diseases and COPD. Variables with *p* < 0.05 were regarded as potential predictors and included in the multivariate regression analysis with age and gender. Age- and gender-adjusted Cox regression analysis with 95%CIs hazard ratio (HR) was used to test occurrence of selected outcomes through days of hospitalization, according to categories of serum calcium on admission comparing the patients in the lowest tertile with the remaining population (tertile I vs tertiles II + III). Relationships between serum calcium and selected inflammation markers (CRP, D-dimer, IL-6) at hospital admission were finally tested using the Spearman’s ranks correlation coefficients (rho) in order to evaluate strength of the associations. For all these analyses a *p* value < 0.05 based on two-sided test was considered statistically significant.

## Results

Table 1 shows main clinical characteristics of the study population, stratified according to tertile categories of serum calcium at hospital admission. All individuals were white Europeans, 35.1% were women, mean age of 67 ± 15 years. Out of 111 patients, 32 died during hospitalization (28.8%) and 39 (35.1%) met the composite outcome of in-hospital death or admission to ICU with need for endotracheal intubation (defined as severe COVID-19). The remaining 72 patients were discharged without need of intensive care during hospitalization (defined as non-severe COVID-19). Among comorbidities, hypertension was the most common (43.1%). At the time of hospitalization mean serum calcium in overall population was 2.1 ± 0.15 mmol/L, with albumin-adjusted calcium median value of 2.2 (2.1–2.27) mmol/L. Eighty-five patients (76.6%) had total serum calcium <2.2 mmol/L ( < 8.8 mg/dL) but none of them showed symptoms suggestive of hypocalcemia, such as paraesthesia, muscle spasms, cramps, tetany, circumoral numbness or seizures. Only 6.7% of patients had levels of 25-OH-D above the cut-off value (≥30 ng/ml) indicated by current guidelines for sufficient vitamin D status [25]. Patients in the lowest tertile of serum calcium at admission had concomitantly the highest median levels of CRP (100.1; 64.6–203.2 mg/l) and IL-6 (57.7; 22.2–101.1 pg/ml). Both these inflammatory parameters had significant reduction trends across increasing tertiles of calcium (*p* < 0.01 for both) (Table 1). Albeit not statistically significant, similar trend was observed for D-dimer and fibrinogen. Median 25-OH-D levels were generally very low in the overall population (12.6; 7.1–18.1 ng/ml), particularly in the subgroup of patients with lower serum calcium (10.6; 5.1–14.6 ng/ml), but without significant differences across tertiles of calcium (Table 1). As well, no differences in age, gender, BMI, creatinine, phosphorus, magnesium and PTH were observed according to serum calcium. Among comorbidities known to affect severity of COVID-19, diabetes mellitus was more prevalent in patients in the lowest tertile of calcium (37 vs 18.8 vs 12.1%, *p* = 0.028), whereas no significant differences were detected in hypertension, cardiovascular diseases and COPD prevalence. In-hospital administration of hydroxychlorochine, antivirals and tocilizumab was driven by local practice and resulted not dissimilar between subgroups. Finally, prevalence of NIV requirement (42.3%), in-hospital mortality (45.7%) and the composite of in-hospital mortality or ICU admission (50%) were consistently higher in the lowest tertile of serum calcium and significantly different with increasing categories of calcium (*p* < 0.05 for all).

**Table 1:** Main clinical features of the study group (*n* = 111) by tertile categories of serum calcium at hospital admission

	*Overall population*	*I tertile* ≤ *2.02* *mmol/L* *(n* = *46)*	*II tertile* *2.02–2.15* *mmol/L* *(n* = *32)*	*III tertile* ≥ *2.15* *mmol/L* *(n* = *33)*	*p value*^*d*^
*Age (years)*	67 ± 15	69.9 ± 13.6	65.8 ± 15.2	65.4 ± 17.1	0.345
*Women*	39 (35.1)	15 (32.6)	10 (31.2)	14 (42.4)	0.592
*BMI (Kg/m*^*2*^*)*	24.6 ± 3.9	24.6 ± 4.3	25.7 ± 4.0	23.5 ± 2.9	0.112
*Serum Calcium (mmol/L)*	2.1 ± 0.15	1.95 ± 0.07	2.1 ± 0.03^c^	2.27 ± 0.1^a,b^	<0.001
*Albumin (g/dl)*	3.4 ± 0.5	3.1 ± 0.4	3.5 ± 0.5^c^	3.7 ± 0.4^a,b^	<0.001
*Albumin-adjusted calcium (mmol/L)*	2.2 (2.1–2.27)	2.1 (2.08–2.17)	2.2 (2.13–2.27)^c^	2.33 (2.23–2.4)^a,b^	<0.001
*PTH (ng/L)*	71 (51.6–93.1)	93.5 (65.7–516)	70.0 (50.4–84.1)	68.2 (54.9–88.1)	0.342
*Serum Phosphorus (mmol/L)*	0.99 ± 0.31	0.92 ± 0.28	1.03 ± 0.25	1.03 ± 0.08	0.087
*Serum Magnesium (mmol/L)*	0.86 (0.79–0.93)	0.87 (0.75–0.93)	0.86 (0.79–0.91)	0.84 (0.79–0.95)	0.986
*25-OH-vitamin D (ng/ml)*^*e*^	12.6 (7.1–18.1)	10.65 (5.1–14.6)	12.8 (9.0–22.5)	13.4 (9.0–19.6)	0.081
*Serum Creatinine (mg/dl)*^*f*^	0.9 (0.71–1.11)	0.92 (0.70–1.14)	0.89 (0.71–1.08)	0.88 (0.76–1.12)	0.942
*eGFR (ml/min/1.73* *m)*^*2*^	83.7 (63.0–99.4)	82.9 (61.9–105.6)	87.9 (69.6–104.4)	80.9 (62.9–92.8)	0.778
*CRP (mg/L)*	83.05 (35.5–141.8)	100.1 (64.6–203.2)	63.8 (22.4–122.7)	40.4 (4.2–142.1)^a^	0.008
*Procalcitonin (ng/ml)*	0.07 (0.04–0.190)	0.105 (0.04–0.350)	0.06 (0.048–0.135)	0.07 (0.023–0.130)	0.114
*IL-6 (pg/ml)*	31.4 (13.1–73.5)	57.7 (22.2–101.1)	30.85 (14.32–78.6)	14.2 (6.5–31.8)^a^	<0.001
*D-dimer (ng/ml)*	1018 (545.2–1675)	1315 (747.5–1641)	839 (430.5–1689)	836 (485–1923.5)	0.101
*Fibrinogen (mg/dl)*	592.5 ± 218.8	620.3 ± 233.8	585.8 ± 187.5	561.1 ± 229.8	0.535
*Time between symptoms onset and hospital admission (days)*	5.0 (1–8)	4.0 (1.0–7.0)	6.0 (0.7–8.2)	5.5 (2.0–9.0)	0.499
*Length of stay (days)*	16 (11.7–22.2)	16 (12.0–21.5)	15 (11.0–25.7)	15 (10.0–22.0)	0.813
*Hypertension*	47 (43.1)	21 (46.7)	13 (41.9)	13 (39.4)	0.831
*Diabetes Mellitus*	27 (24.3)	17 (37.0)	6 (18.8)	4 (12.1)	0.028
*Cardiovascular diseases*	35 (32.1)	17 (37.8)	9 (29.0)	9 (27.3)	0.594
*COPD*	8 (7.3)	3 (6.7)	3 (9.7)	2 (6.1)	0.809
*NIV therapy*	20 (25.3)	11 (42.3)	4 (16.0)	5 (17.9)	0.062
*ICU admission*	17 (15.3)	7 (15.2)	6 (18.8)	4 (12.1)	0.711
*In-hospital death*	32 (28.8)	21 (45.7)	7 (21.9)	4 (12.1)	0.003
*Death or ICU*	39 (35.1)	23 (50.0)	10 (31.3)	6 (18.2)	0.012
*Hydroxychloroquine therapy*	98 (90.7)	37 (84.1)	29 (93.5)	32 (97.0)	0.152
*Antiviral drugs therapy*	79 (73.1)	31 (70.4)	25 (80.6)	23 (69.7)	0.572
*Tocilizumab therapy*	21 (19.6)	9 (20.9)	8 (25.8)	4 (12.1)	0.378

Continuous variables are expressed as means ± SD or median (interquartile range). Discrete traits are expressed as *n* (%)

Abbreviations as described in the text

^a^ < 0.05 vs I tertile; ^b^ < 0.05 vs II tertile; ^c^ < 0.05 vs I tertile: post-hoc test for multiple comparisons

^d^Test for linear trend (continuous variables) or for proportions (discrete traits)

^e^Conversion factor for 25-hydroxyvitamn D from ng/ml to nmol/L is 2.496

^f^Conversion factor for serum creatinine from mg/dl to µmol/L is 88.4

**Fig. 1:**
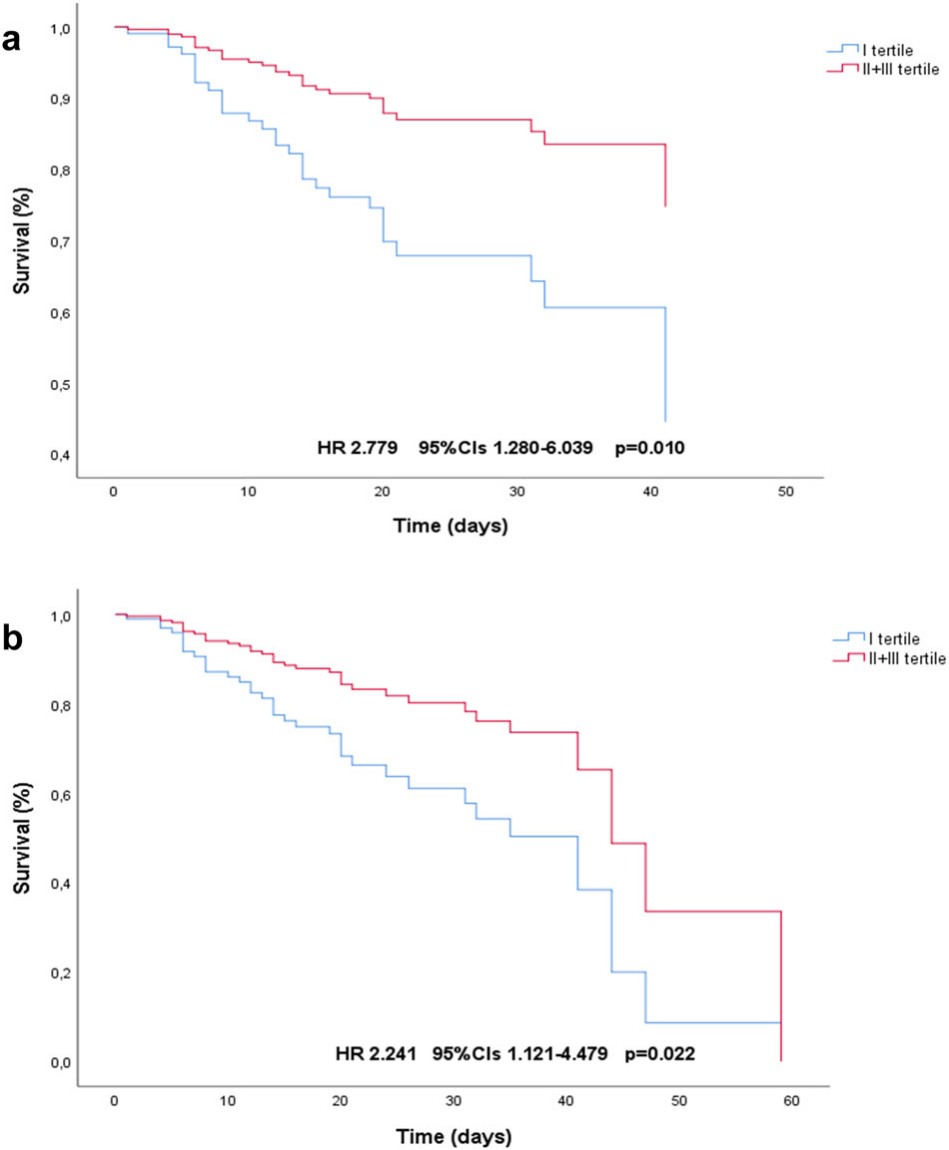
Cox regression analysis curves show significant difference in survival from in-hospital death (**a**) and severe COVID-19 (**b**) through days of hospitalization for patients stratified according to tertile categories of serum calcium at admission (tertile I vs tertile II + III)

**Fig. 2:**
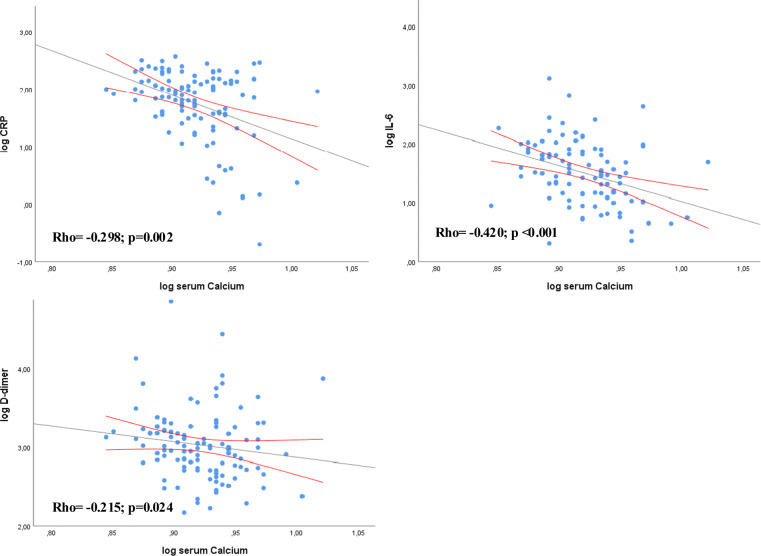
Scatterplotts of bivariate correlation analysis show significant inverse associations between serum calcium and inflammatory markers (CRP, IL-6, D-dimer) collected at admission

In the univariate regression analysis, patient characteristics emerged as potential predictors of the composite primary outcome (in-hospital death or ICU admission) were age, 25-OH-D, CRP, procalcitonin, IL-6, creatinine, time from symptoms onset to hospitalization, diabetes mellitus, serum calcium values and category of serum calcium (tertile I vs tertile II + III), as shown in Table 2. The subsequent multivariable analysis, adjusted for age and gender, confirmed significant associations with severe COVID-19 disease only for CRP, procalcitonin, vitamin D and serum calcium, with an OR of 2.82 (95%CI 1.209–6.613; *p* = 0.016) for patients in the I tertile (serum calcium ≤2.02 mmol/L) to meet the primary outcome compared to the remaining population (Table 2). Accordingly, in the age- and gender-adjusted Cox regression analysis shown in Fig. 1, stratified according to calcium categories (tertile I vs tertile II + III), patients in the lowest tertile had higher risk of in-hospital death (HR 2.779; 95%CI 1.280–6.039; *p* = 0.010) and severe COVID-19 (HR 2.241; 95%CI 1.121–4.479; *p* = 0.022). Finally, as shown in Fig. 2, bivariate correlation analysis between initial serum calcium values and the selected inflammatory markers (CRP, IL-6, D-dimer) revealed, as expected, a significant negative association in all the performed tests (*p* < 0.05 for CRP and D-dimer; *p* < 0.001 for IL-6). Serum calcium was strongly and positively associated with circulating 25-OH-D (*p* = 0.006), whereas no significant association (*p* = 0.219) was found between calcium and PTH levels at the time of admission.

**Table 2:** Univariate and multivariate analysis of predictors for in-hospital death or ICU admission in all individuals (*n* = 111)

		*Univariate analysis* *OR (95%CI)*	*p value*	*Multivariate analysis*^*a*^ *OR (95%CI)*	*p value*
*Age (years)*		1.054 (1.021–1.088)	0.001		
*Gender*	*Female*	1 (ref)	0.589		
	*Male*	0.800 (0.356–1.798)			
*BMI (Kg/m*^*2*^*)*		1.037 (0.926–1.162)	0.530		
*Serum Calcium (mmol/L)*		0.382 (0.183–0.798)	0.010	0.444 (0.212–0.930)	0.032
*Categories of initial serum calcium*	*II-III tertile*	1 (ref)	0.007	1 (ref)	0.016
	*I tertile*	3.062 (1.365–6.869)		2.828 (1.209–6.613)	
*Serum Phosphorus (mmol/L)*		0.729 (0.461–1.153)	0.176		
*Serum Magnesium (mmol/L)*		0.611 (0.177–2.112)	0.436		
*PTH (ng/L)*		1.007 (0.997–1.018)	0.192		
*25-OH-vitamin D (ng/ml)*		0.878 (0.805–0.957)	0.003	0.892 (0.819–0.971)	0.008
*Serum Creatinine (mg/dl)*		4.006 (1.010–15.889)	0.048	3.333 (0.762–14.584)	0.110
*CRP (mg/L)*		1.010 (1.005–1.016)	<0.001	1.009 (1.003–1.015)	0.002
*Procalcitonin (ng/ml)*		4.702 (1.221–18.105)	0.024	3.914 (1.071–14.307)	0.039
*IL-6 (pg/ml)*		1.007 (1.000–1.013)	0.044	1.005 (0.999–1.012)	0.109
*D-dimer (ng/ml)*		1.000	0.308		
*Fibrinogen (mg/dl)*		1.001 (0.999–1.003)	0.183		
*Time from symptoms onset to ER admission (days)*		0.886 (0.775–0.967)	0.011	0.904 (0.804–1.017)	0.092
*Hypertension*	No	1 (ref)	0.985		
	Yes	1.008 (0.453–2.244)			
*Diabetes Mellitus*	No	1 (ref)	0.040	1.717 (0.664–4.443)	0.265
	Yes	2.542 (1.046–6.177)			
*Cardiovascular diseases*	No	1 (ref)	0.360		
	Yes	1.478 (0.640–3.412)			
*COPD*	No	1 (ref)	0.826		
	Yes	1.182 (0.267–5.244)			

Abbreviations as described in the text

^a^Adjusted for age and gender

## Discussion

In this study we reported a significant association of low total serum calcium levels at admission with adverse prognosis in patients hospitalized for COVID-19. Due to the low mean calcium values (2.1 ± 0.15 mmol/L) found in the study population, the main clinical characteristics analyzed are shown in Table 1 stratified according tertile categories of total serum calcium, while, in Table 3 is shown the comparison of the main clinical features of the study group stratified according to the presence of hypocalcemia (total serum calcium <2.2 mmol/L) or not. In line with other previous reports on patients requiring hospitalization for COVID-19, we found a high prevalence of hypocalcemia at hospital admission, with 76.6% of subjects having total serum calcium <2.2 mmol/L (<8.8 mg/dL) and a significant inverse correlation between calcium levels and biomarkers of severe inflammatory state [3, 5, 7], while other Authors have already shown that hypocalcemia is an independent risk factor for hospitalization in patients with SARS-CoV-2 infection [4]. Liu et al. reported in their study a similar, but slightly lower, prevalence of hypocalcemia in severe COVID-19 patients and this is probably due to the different method used in assessing serum calcium levels, according to albumin-adjusted instead of total serum calcium [5]. This latter point may be of crucial importance, with several studies on large case series supporting the use of total serum calcium in assessing calcium levels because of its greater accuracy and better concordance with ionized calcium status [26–28]. Nevertheless, we reported in Table 1 median (IQR) albumin-adjusted calcium values for each tertile and we performed bivariate correlation analysis between total and albumin-adjusted calcium that showed, as expected, a strong positive relationship (rho 0.721, *p* < 0.001). Moreover, we repeated the analysis displayed in Table 1 across tertile categories of albumin-adjusted calcium, with results confirming those of the previous analysis. The prognostic value of low calcium levels in critically ill patients has been largely investigated [17–20]. Interestingly, a large retrospective study found a U-shaped association curve between initial calcium levels and mortality in critical patients receiving intensive care, with a nadir of mortality for calcium values within the normal range [29]. These observations support the hypothesis that, in critically ill patients, any changes in serum calcium concentrations may reflect the severity of the underlying disease, without necessarily implying a causative effect toward the increased mortality. Accordingly, the exact mechanisms underlying the impairment of calcium homeostasis in critically ill patients has not yet been fully understood, potentially including intracellular accumulation of calcium ions, impaired parathyroid function and parathyroid hormone peripheral resistance, or impaired metabolism and action of vitamin D [15, 17]. A key role in these processes appears to be played by proinflammatory cytokines, whose blood levels correlate directly with disease severity and inversely with calcium levels [21]. Accordingly, in our COVID-19 population, bivariate correlation analysis revealed significant negative association between initial total calcium and IL-6 circulating levels, which has been shown to be produced by alveolar macrophages and dendritic cells in response to SARS-CoV-2 infection of the respiratory epithelium [30, 31]. In addition, IL-1β and IL-6 have been demonstrated to up-regulate the calcium-sensing receptor (CaSR) on parathyroid glands and kidneys, leading in turn to reduced circulating PTH and 1,25-(OH)_2_-vitamin D, and to down-regulate PTH receptors expression on target tissues, inducing a sort of peripheral tissues resistance to PTH signaling [21]. In our COVID-19 population, in-hospital mortality was remarkably high (28.8%), while 35.1% of patients required ICU admission for endotracheal intubation. In accordance with previous studies on calcium metabolism and COVID-19 [3, 5], we found that prevalence rates of NIV requirement, in-hospital death, and the composite of in-hospital death or ICU admission, were significantly higher among patients in the lowest tertile of serum calcium (42.3%, 45.7% and 50% of the total population, respectively). In addition, median values of CRP and IL-6 were higher in these patients, whereas significantly decreased across tertiles of serum calcium (*p* < 0.01 for both, tertile III vs tertile I) (Table 1). As expected PTH levels were also inversely related to serum calcium, even though the trend observed across tertiles was not significant. This can be attributable, at in least in part, to the small number of patients for whom PTH levels were available (just 26 subjects). Otherwise, the effect of parathyroid dysfunction and peripheral resistance to PTH, both induced by cytokines [15, 17, 21], are other potential explanations for hypocalcemia occurrence, as reported in literature [32], and for PTH having been resulted not significantly dissimilar between categories of calcium. Among other conditions well-recognized to be related with poorer outcome of COVID-19, diabetes mellitus was significantly more prevalent in the lowest compared with highest tertile of calcium (37 vs 12.1%, *p* = 0.028). Intriguingly, diabetes is an established risk factor for respiratory infections, as well as impairment of calcium homeostasis is frequently observed in patients with severe infectious diseases [15, 17]. The mechanisms through which diabetes mellitus can worsen COVID-19 course have not yet been fully clarified and several factors could be involved, including a state of chronic low-grade inflammation, which could predispose to the cytokine storm. IL-6 could play an important role in these processes because, in addition to being involved in the acute phase of inflammatory response, its concentrations significantly increased in those conditions characterized by chronic subclinical inflammation, such as metabolic disorders, overweight and cardiovascular diseases. In this sense, one may hypothesize that the increased level of IL-6 - or other inflammatory mediators involved in the impaired glucose regulation state – can play a role linking together diabetes mellitus and hypocalcemia, as emerged in our population [33, 34]. This study also highlighted high prevalence of impaired vitamin D status among patients hospitalized for COVID-19, with only 6.7% of subjects reporting 25-OH-vitamin D levels ≥30 ng/ml [25]. Although median 25-OH-vitamin D levels were remarkably low (12.6; 7.1–18.1 ng/ml)—and particularly in patients with lower total calcium levels (10.6; 5.1–14.6 ng/ml)—differences observed among tertiles of calcium were not statistically significant. When analyzing metabolic factors associated with critical in-hospital course of COVID-19, we observed significant relationships for both total serum calcium and 25-OH-vitamin D levels detected on admission. In particular, patients with total serum calcium ≤2.02 mmol/L had roughly threefold increased probability of being included in the group with adverse in-hospital outcomes than those with higher calcium levels (Table 2). The Cox regression analysis, which additionally provides information about temporal progression of the selected outcomes (Fig. 1), confirmed that patients in the bottom tertile of calcium (blue line) had greater risk of death (HR 2.779; 95%ICs 1.280–6.039, *p* = 0.01) and severe COVID-19 outcome (HR 2.241; 95%ICs 1.121–4.479, *p* = 0.02) during hospital stay when compared with others (red line), confirming, thus, the hypothesis that the lower the calcium levels at hospital admission, the worse the outcomes during the hospitalization for COVID-19. Regarding vitamin D status, since no differences according to calcium levels were detected (Table 1), we might speculate that the observed relationship with severity of COVID-19 is unrelated to the role that vitamin D exerts in calcium homeostasis regulation, rather reflecting its action on the modulation of the immune response and production of proinflammatory cytokines [35]. In accordance, vitamin D receptor has been identified in the respiratory epithelium and alveolar macrophages, and its active metabolite calcitriol can be additionally produced by local macrophages themselves and dendritic cells [36]. Beyond its origin, calcitriol could negatively modulate, by both endocrine and autocrine/paracrine mechanisms, the production of proinflammatory cytokines and increase the expression of antimicrobial proteins (e.g., cathelicidin and β2 defensin), as showed by various in-vitro studies in different cell lines [35, 37, 38]. When putting such findings into the clinical context, a recent meta-analysis of 25 randomized controlled trials reported that vitamin D supplementation may exert a protective role against acute respiratory tract infections [39]. In this sense, it would be of interest to properly explore these findings in a population with SARS-CoV-2 infection, since remarkable prevalence of hypovitaminosis D has been observed in these patients [35, 38], characterizing, together with hypocalcemia and impaired PTH response, the osteo-metabolic phenotype recently described in these patients [7], as well as an inverse relationship between vitamin D levels and mortality for COVID-19 has been reported by several Authors from different countries, conferring to the question particular relevance [40–42].

**Table 3:** Main clinical features of the study group (*n* = 111) at hospital admission stratified by total serum calcium values < 2.2 mmol/L (hypocalcemia) or ≥ 2.2 mmol/L

	*Overall population*	*Total serum calcium* < *2.2* *mmol/L*	*Total serum calcium* ≥ *2.2* *mmol/L*	*p value*
*Age (years)*	67 ± 15	68 ± 14	64 ± 18	0.195
*PTH (ng/L)*^*a*^	71 (51.6–93.1)	71.4 (51.5–99.0)	70.6 (52.6–98.2)	0.996
*25-OH-vitamin D (ng/ml)*^*a*^	12.6 (7.1–18.1)	12.3 (6.5–18.4)	13.2 (7.2–18.0)	0.475
*Serum Creatinine (mg/dl)*	0.9 (0.71–1.11)	0.91 (0.71–1.14)	0.88 (0.76–1.04)	0.805
*CRP (mg/L)*	83.05 (35.5–141.8)	88.8 (39.3–149.2)	58.5 (3.9–140.6)	0.123
*Procalcitonin (ng/ml)*	0.07 (0.04–0.190)	0.07 (0.04–0.27)	0.07 (0.02–0.145)	0.130
*IL-6 (pg/ml)*	31.4 (13.1–73.5)	35.2 (20.4–81.6)	12.4 (5.6–35.5)	0.001
*D-dimer (ng/ml)*	1018 (545.2–1675)	1073 (580.5–1703)	824 (501–1567)	0.280
*Length of stay (days)*	16 (11.7–22.2)	16 (12–23)	14 (9–22)	0.442
*In-hospital death*	32 (28.8)	29 (34.1)	3 (11.5)	0.028
*Death or ICU*	39 (35.1)	34 (40)	5 (19.2)	0.062

Differences in normally distributed continuous variables between categories of serum calcium were assessed using the Student *T* test, whereas the Mann–Whitney U test was used in case of skewed variables. Differences in proportions of discrete traits were assessed using *χ*² test and Fisher’s exact test

Continuous variables are expressed as means ± SD or median (interquartile range). Discrete traits are expressed as *n* (%)

^a^PTH and 25-OH-vitamin D values were only available for 26 and 77 patients, respectively

In this clinical work, we focused on the role of hypocalcemia, defined as total serum calcium <2.2 mmol/L, in assessing the risk for adverse outcomes in hospitalized patients with COVID-19. This study gives an overview of the possible mechanisms involved in the association between calcium levels and inflammatory biomarkers, considering not only vitamin D and parathyroid hormone levels, but also other clinical conditions, such as diabetes mellitus and cardiovascular disease [33, 34]. This point is one of the primary strengths of our work and the most significant distinction from previous studies [3–5]. The main limitation of our study lies in the retrospective data collection and in its monocentric nature. Moreover, these observations derive from a fully unselected population and data were extract from hospital clinical records. Indeed, due to a number of technical reasons related to hospitalization, PTH and 25-OH-vitamin D values, two of the main parameters of mineral metabolism, were unfortunately not available for all the patients included in the analysis.

In conclusion, our observational data confirmed remarkably high prevalence of hypocalcemia (total serum calcium <2.2 mmol/L) at hospital admission in patients with COVID-19. Being in the lowest calcium levels tertile was associated with roughly 3-fold increased probability of critical in-hospital course compared with other patients. Total calcium levels were also significantly and inversely related with acknowledged inflammatory biomarkers of severe SARS-CoV-2 infection. Evaluation of total calcium, alongside other already established risk factors, contribute to stratify patients admitted with COVID-19 and improve their in-hospital management.
